# Current Practice Patterns in Microvascular Decompression for Trigeminal Neuralgia: Results From an International Survey in Central Europe

**DOI:** 10.7759/cureus.100133

**Published:** 2025-12-26

**Authors:** Johann Klein

**Affiliations:** 1 Neurosurgery, Helios Dr. Horst Schmidt Kliniken, Wiesbaden, DEU

**Keywords:** ‏facial pain, jannetta procedure, microvascular decompression (mvd), survey, trigeminal neuralgia

## Abstract

Background* *

Trigeminal neuralgia is a facial pain disorder, most often caused by neurovascular contact at the trigeminal root. In cases refractory to pharmacotherapy, microvascular decompression (MVD) is an established treatment. Since its description, various modifications have been introduced. The extent to which current practice deviates from the original approach remains unclear. This study aimed to assess current strategies in MVD for trigeminal neuralgia.

Methods

A cross-sectional survey study was conducted by distributing a web-based 14-item questionnaire to neurosurgeons with expertise in MVD from Germany, Austria, Switzerland, Italy, and Luxembourg. The link to the survey was shared between May and August 2025 and was not made public. To avoid institutional clustering, only one practitioner per institution was invited to participate anonymously.

Results

Of 123 neurosurgeons invited, 63 completed the questionnaire (response rate 51.2%). Around 59/63 respondents (93.7%) do not consider an age limit for MVD. Surgery is most commonly performed in the supine (29/63 respondents, 46.0%) or lateral (25/63 respondents, 39.7%) position, either purely microscopically (41/63 respondents, 65.1%) or with endoscopic assistance (19/63 respondents, 30.2%). Neuromonitoring is regularly used by 45/63 practitioners (71.4%). Trepanation is usually performed either osteoplastically or osteoclastically with Palacos cranioplasty (23/63 respondents, 36.5% each). Teflon is the most frequently used interposition material (47/63 respondents, 74.6%). Around 41/63 participants (65.1%) never sacrifice the superior petrosal vein. Only five of 63 (7.9%) would generally refrain from MVD if no neurovascular contact is evident on radiological examination.

Conclusions

This survey demonstrates current practice patterns in MVD for trigeminal neuralgia in Central Europe. While some principles of the original technique remain widely adopted, notable changes in operative strategy are apparent, highlighting areas for future consensus-building and research and informing operative decision-making.

## Introduction

Trigeminal neuralgia is a painful disorder characterized by brief paroxysms in the distribution area of one or several branches of the trigeminal nerve [1]. The pain attacks are most often described as electric shock-like and are typically triggered by innocuous stimuli such as chewing, touch, brushing teeth, eating, talking, and cold wind [2]. Depending on the etiology, trigeminal neuralgia is classified as classical if caused by vascular compression of the trigeminal root, secondary if attributed to multiple sclerosis, tumor, or other cause, or idiopathic if no abnormalities are detected upon radiological and electrophysiological examination [3]. Pharmacotherapy is the initial treatment, with carbamazepine and oxcarbazepine being the most effective drugs [4]. While the initial response rates are 98% and 94%, respectively, adverse events are frequent, causing 27% (carbamazepine) and 18% (oxcarbazepine) of patients to discontinue treatment [5].

In patients with classical trigeminal neuralgia who cannot be satisfactorily managed with pharmacotherapy, microvascular decompression (MVD), initially described by Peter Jannetta, is the surgical treatment of choice [6]. The operation includes a retrosigmoid trepanation and dissection of the trigeminal nerve in the cerebellopontine angle to separate the offending vessel from the nerve. A prospective study with independent pre- and postoperative evaluation revealed a success rate of 86% [7]. Retrospective analyses consistently show a high proportion of favorable outcomes in the long term [8]. After decades of experience, the Jannetta group published their surgical method, which defined, among other steps, the placement of the patient in the lateral position, retrosigmoid craniectomy, insertion of a retractor, coagulation and division of the petrosal veins, mobilization of the offending vessel, interposition with a Teflon felt, watertight dural closure, and cranioplasty with methyl methacrylate or wire mesh as crucial elements of the technique [9].

Since then, significant technological advances have shaped the field of neurosurgery, including improvements in operating microscopes, neuroendoscopy, neuronavigation, and operating techniques such as sling retraction or the use of alternative interposition materials. An increasing number of elderly patients, as well as progress in anesthesiology and peri-procedural care, raises the question of performing surgery in patients once deemed too old. While a multitude of variations to the Jannetta procedure grounded in these developments were reported, comparative studies are scarce and often not feasible. Furthermore, it is unknown how many of these variations are adopted by the neurosurgical community and how far MVD, as it is performed today, departs from the principles defined by Peter Jannetta.

This study aimed to determine current practice patterns and operative standards in MVD for trigeminal neuralgia among neurosurgeons in several Central European countries and discuss them in the context of the available literature. It was previously posted to the Research Square preprint server on September 4, 2025, and presented as a speech at the 2025 Conference of the Vascular Section of the German Society of Neurosurgery on November 7, 2025.

## Materials and methods

Study design

This cross-sectional survey study was designed as a descriptive assessment of current practice rather than a hypothesis-driven comparative analysis. A web-based questionnaire was developed using an online platform (www.survio.com). It consisted of 14 questions with one item per page and was written in German (Appendix 1). Each question had to be answered to submit the questionnaire. No questions on personal data (such as age, sex, location, duration of occupation, or number of surgeries performed) were included to protect anonymity in a relatively small specialist cohort.

The survey was distributed among neurosurgeons in Germany, Austria, Luxembourg, and the German-speaking parts of Switzerland and Italy (South Tyrol), who have expertise in MVD. To determine the appropriate institutions and practitioners, a list was created containing all neurosurgical departments in the relevant areas, using information from the respective neurosurgical societies. Departments with a focus on spine surgery were excluded, as were those for which no sufficient expertise in MVD could be assumed based on institutional service descriptions and published case portfolios. Appropriate addressees within the departments were determined by research (including information on the practitioners’ or their employers’ websites and publications), acquaintance, or inquiry. If no practitioner involved in the management of trigeminal neuralgia and MVD could be determined, the head of the department was addressed. Only one neurosurgeon per institution was invited to participate. A link to the survey was distributed either in person at neurosurgical events or via email between May 27 and August 11, 2025, briefly describing the survey's aims and stating the number of items and the estimated time required for completion. Each invitee was addressed individually. No mass emails were sent, and the link to the survey was not made public. No incentive, financial or otherwise, was offered for participation. No routine reminders were sent; however, a follow-up message could be sent in individual cases, primarily if an automated out-of-office reply was received in response to the initial email.

The responses recorded could not be assigned to the participants through cookies, and no IP addresses of the client computers were visible to the author.

Statistical analysis

Descriptive statistics are presented as percentages and absolute numbers, as appropriate. Sums exceeding 100% are due to rounding. No inferential statistics were employed.

Ethics and reporting

The ethics committee of the Medical Association of Hesse waived the need for informed consent. Completion of the questionnaire was considered implied consent. The reporting of this study follows the Checklist for Reporting Results of Internet E-Surveys (CHERRIES).

## Results

Six neurosurgeons were invited to participate in person, and 117 via email. Of 123 neurosurgeons invited, 63 completed the survey (response rate 51.2%).

The majority of respondents (59/63, 93.7%) do not see an age limit for MVD indication. A microsurgical approach is most frequently employed (41/63 respondents, 65.1%), followed by microsurgery with endoscopic assistance (19/63 respondents, 30.2%), whereas fully endoscopic or exoscopic approaches are rarely used. Around 29/63 participants (46.0%) reported preferring a supine position, and 25/63 (39.7%) a lateral/park-bench position.

Neuronavigation is never or rarely used by 40/63 participants (63.5%), while 16/63 (25.4%) reported regular use. Neuromonitoring is routinely applied by 45/63 respondents (71.4%). Most respondents perform either an osteoplastic craniotomy or an osteoclastic craniectomy followed by cranioplasty, using Palacos or another bone replacement.

Fixed retraction is generally or often applied by 33/63 survey subjects (52.4%), whereas 25/63 (39.7%) avoid or limit its use. The superior petrosal vein is preserved by most (41/63, 65.1%), but 18/63 (28.6%) sacrifice it when necessary. Teflon is the most common interposition material (47/63, 74.6%), while muscle, other materials, and transposition without interposition were less frequently reported.

Most respondents regularly secure the graft with fibrin glue (40/63, 63.5%). Of note, all six neurosurgeons who indicated using muscle as an interposition material reported applying fibrin glue. Watertight dural closure is pursued by the majority. Lumbar drains are rarely used.

When asked about MVD in trigeminal neuralgia secondary to multiple sclerosis, 35/63 contributors (55.6%) would consider surgery if MRI showed neurovascular contact, nine of 63 (14.3%) would generally perform MVD, while 19/63 (30.2%) would not. In idiopathic trigeminal neuralgia with the absence of neurovascular contact on imaging, 42/63 (66.7%) sometimes offer MVD after shared decision-making, 16/63 (25.4%) generally do, and five of 63 (7.9%) do not. Table 1 provides a detailed account of questions and responses.

**Table 1:** Survey items and distribution of responses (n = 63) MRI: magnetic resonance imaging; MS: multiple sclerosis; MVD: microvascular decompression; NVC: neurovascular contact; TN: trigeminal neuralgia

Item	Question	Response Options	n (%)
1	Age limit for MVD indication	None	59 (93.7)
		Yes ≥80 years	4 (6.4)
		Yes, 75–79 years	0 (0.0)
		Yes, 70–74 years	0 (0.0)
2	Approach	Microsurgical	41 (65.1)
		Microsurgical + endoscopic	19 (30.2)
		Fully endoscopic	1 (1.6)
		Exoscopic	1 (1.6)
		Varies	1 (1.6)
3	Positioning	Supine	29 (46.0)
		Lateral/park bench	25 (39.7)
		Prone	4 (6.4)
		Sitting/half-sitting	1 (1.6)
		Varies	4 (6.4)
4	Use of neuronavigation	No/rarely	40 (63.5)
		Yes, always/often	16 (25.4)
		Sometimes	7 (11.1)
5	Use of neuromonitoring	Yes	45 (71.4)
		No	13 (20.6)
		Sometimes	5 (7.9)
6	Type of trepanation	Osteoplastic	23 (36.5)
		Osteoclastic + Palacos, bone cement, etc.	23 (36.5)
		Osteoclastic, no cranioplasty	1 (1.6)
		Osteoclastic + allogenic material (mesh, etc.)	2 (3.2)
		Osteoclastic + bone dust	7 (11.1)
		Varies	7 (11.1)
7	Fixed retraction	Yes, always/often	33 (52.4)
		No/only during approach	25 (39.7)
		Varies	7 (11.1)
8	Sacrifice of the superior petrosal vein	No, never, or only upon hemorrhage	41 (65.1)
		Yes, if necessary	18 (28.6)
		Yes, generally/often	4 (6.4)
9	Interposition material	Teflon	47 (74.6)
		Muscle	6 (9.5)
		Other	4 (6.4)
		Transposition/sling retraction without interposition	6 (9.5)
10	Fixation with fibrin glue	Yes, always/usually	40 (63.5)
		No/rarely	16 (25.4)
		Sometimes	7 (11.1)
11	Watertight dural closure	Yes, absolutely	53 (84.1)
		No, not imperatively	10 (15.9)
12	Routine lumbar drain placement	No	60 (95.2)
		Yes, before surgery	3 (4.8)
		Yes, after surgery	0 (0.0)
13	MVD in TN with MS	Yes, if NVC on MRI	35 (55.6)
		Yes	9 (14.3)
		No	19 (30.2)
14	MVD in TN without NVC on MRI	Sometimes (shared decision)	42 (66.7)
		Yes	16 (25.4)
		No	5 (7.9)

Several respondents provided additional comments via email after completing the survey, thereby identifying themselves. While these comments were generally not linked to survey responses, one clarification led to the reclassification of a response in item 9, where an answer originally marked “Teflon” was reassigned to “Transposition without interposition material” based on the participant’s detailed explanation.

## Discussion

This study provides an overview of current views on both indications for MVD and operative techniques among a selected group of neurosurgeons in Central Europe. It is, to the author’s knowledge, the first to systematically examine intraoperative strategies and technical details in MVD for trigeminal neuralgia. A prior survey from the United States was distributed to all members of the American Association of Neurological Surgeons and exclusively addressed preoperative decision-making, without exploring operative conduct [10]. By focusing on operative nuances, the present study provides novel insight into how the procedure has evolved.

Patient selection

While the incidence of trigeminal neuralgia increases with age, so does the fear of surgical complications. Therefore, the finding that the vast majority of the respondents see no age limit for the indication for MVD is particularly noteworthy. Concerning elderly patients in reduced general condition, it should be pointed out that percutaneous rhizotomies may present a reasonable alternative to MVD [11]. The same applies to trigeminal neuralgia secondary to multiple sclerosis and idiopathic trigeminal neuralgia [12]. The use of MVD in treating these conditions is unclear. A seminal study by Truini et al. suggested a double-crush mechanism in trigeminal neuralgia secondary to multiple sclerosis, with both inflammatory demyelination and neurovascular compression contributing to the manifestation of the disorder concurrently, implying a role for MVD in its treatment [13]. Others, however, have challenged this observation but still recognized that MVD should be considered in this patient population if neurovascular contact with morphological changes is established [14]. Overall, MVD may be appropriate in trigeminal neuralgia secondary to multiple sclerosis, although the success rates appear to be lower compared to classical trigeminal neuralgia [15]. In this survey, nine of 63 participants (14.3%) stated they generally consider MVD in patients with multiple sclerosis, while 35/63 (55.6%) do so if neurovascular contact is demonstrated.

Only five of 63 respondents (7.9%) indicated that they don’t consider MVD if no neurovascular contact is shown on magnetic resonance imaging (MRI). This finding likely reflects the observation that despite advances in modern radiological examinations, false-negative results are possible. In one study, 14 out of 17 patients with trigeminal neuralgia, in whom the preoperative MRI was negative for neurovascular relationship, were found to have a vascular contact to the trigeminal nerve during surgery [16].

Operative technique

While some operative details have largely been maintained since Jannetta’s description, other aspects show greater variation, for example, positioning or trepanation. In some regards, notably the handling of the superior petrosal vein, Jannetta’s approach has largely been abandoned, as only four of 63 practitioners (6.4%) routinely sacrifice it, and 41/63 (65.1%) will never do so unless hemorrhage forces them to. In the past, some authors argued that obliteration of veins in the cerebellopontine angle is safe. In a recent review, however, Joswig et al. undertook a thorough appraisal of reports on superior petrosal vein sacrifice and its ensuing complications. They concluded that the maneuver poses too significant a risk and should be considered obsolete [17].

Nearly one-third of participants use neuroendoscopy, albeit only one practitioner (1.6%) indicated performing fully endoscopic MVD. Proponents of endoscopy cite improved visualization of anatomic structures and, in the case of fully endoscopic approaches, less need for tissue dissection compared with purely microsurgical approaches [18]. However, comparative studies failed to detect differences in clinical outcomes [19]. Neuronavigation is regularly used in MVD by one in four respondents. Purported advantages include reduced craniotomy size, avoidance of unnecessary opening of mastoid air cells, and reduced incidence of CSF leaks [20]. To avoid complications such as postoperative hearing loss and facial palsy, the majority of neurosurgeons in this survey indicated regularly using neuromonitoring, which is an established method to improve outcomes in MVD [21]. Damage to the VII and VIII cranial nerves during MVD may result from direct manipulation, vasospasm, forceful irrigation, CSF egress, acoustic trauma from drilling, or tension injury through retraction of the cerebellum [22]. Although described in MVD for hemifacial spasm rather than trigeminal neuralgia, greater cerebellar retraction depth has been identified as significantly associated with postoperative hearing loss [23]. To avoid this complication, 25/63 neurosurgeons in this survey (39.7%) don’t use fixed cerebellar retraction during MVD or do so only during the approach to the cerebellopontine angle. A minority places a lumbar drain before the surgery to drain CSF and relax the cerebellum. A large MVD series identified the lack of primary dural closure as a significant risk factor for postoperative CSF leak [24]. Accordingly, 53/63 respondents (84.1%) in this survey pursue watertight dural closure, independently of the use of dural sealants or augmentation.

Interposition and transposition

The majority of the participants separate the offending vessel from the nerve using Teflon as an interposition material. Despite high success rates using this technique, Teflon granulomas or adhesions may lead to pain recurrence [25]. To avoid these occurrences, some authors favor transposition of the offending artery without interposition material [26]. In this survey, six of 63 participants (9.5%) indicated attempting transposition over interposition, if feasible. However, comparative studies show no significant differences in outcomes between the two techniques [27]. An additional six respondents (9.5%) use autologous muscle as interposition material. Numerous reports indicate excellent results with muscle grafting [28], while there is no conclusive evidence to favor one interposition material over the other [29]. Notably, all participants using muscle in this study apply fibrin glue to secure the graft, while, overall, 16/63 survey subjects (25.4%) never do so in MVD. This can be interpreted as confirming the observation that Teflon has a higher tendency to adhere to the surrounding tissues than does muscle.

Strengths and limitations

Strengths of the present study include its strict methodology, inviting only one practitioner per institution, and an attempt to make the survey accessible only to neurosurgeons with experience in MVD. The high response rate of more than 50% indicates the relevance and importance of the questions raised. Still, there are limitations. Despite efforts to identify appropriate addressees, there can be no certainty that the respondents are representative of all MVD practitioners. This is especially true for neurosurgeons outside of the geographic region included in this survey, which was limited to German-speaking Central Europe. A self-selection bias is inherently possible in surveys, as those more interested in the subject of the questionnaire may have been more likely to respond. The results do not allow for allocation of the responses to individual levels of experience or treatment outcomes. However, questions about personal qualifications, such as the number of MVD procedures performed, were deliberately omitted to ensure anonymity in a comparatively small neurosurgical cohort and to avoid bias, as the self-assessment of physicians is demonstrably of limited accuracy [30]. Because the survey was designed to ensure complete anonymity, data on individual case volumes and center size could not be collected. This design choice was made to encourage honest reporting and maximize participation. Consequently, subgroup analyses by surgical volume were not possible.

## Conclusions

This study shows that the surgical techniques in MVD have evolved since the procedure was developed by Peter Jannetta. While key principles remain widely adopted, significant changes in intraoperative strategy are evident. These findings may help inform consensus-building and identify areas for further research, including the development of a registry for MVD. Furthermore, for operative steps that lack evidence-based consensus, it can be valuable for practitioners to consider the approach of others, as presented in this article, for their own decision-making. Finally, the author would like to encourage neurosurgeons from other parts of the world to research practice patterns in MVD in their respective regions.

## Appendices

Appendix 1

**Table 2:** Questions and responses in their original wording, translated to English

Item	Question	Response Options	n (%)
1	Do you see an age limit for the indication of microvascular decompression, regardless of comorbidities?	yes: ≥80 years	4 (6.35)
		yes: 75-79 years	0 (0)
		yes: 70-74 years	0 (0)
		no	59 (93.65)
2	How do you usually perform microvascular decompression?	microsurgically	41 (65.08)
		microsurgically with endoscopic assistance	19 (30.16)
		purely endoscopically	1 (1.59)
		exoscopically (Orbeye, etc.)	1 (1.59)
		varies	1 (1.59)
3	In which position do you usually perform microvascular decompression?	supine	29 (46.03)
		lateral/park bench	25 (39.68)
		prone	4 (6.35)
		sitting/semi-sitting	1 (1.59)
		varies	4 (6.35)
4	Do you use neuronavigation to plan trepanation?	yes, always or often	16 (25.40)
		no, never or rarely	40 (63.49)
		sometimes	7 (11.11)
5	Do you use neuromonitoring (facial nerve MEP, AEP) during microvascular decompression?	yes	45 (71.43)
		no	13 (20.63)
		sometimes	5 (7.94)
6	How do you perform trepanation for microvascular decompression?	osteoplastic	23 (36.51)
		osteoclastic with cranioplasty using Palacos, bone replacement, or similar	23 (36.51)
		osteoclastic with re-insertion of bone dust	7 (11.11)
		osteoclastic with allogenic reconstruction (mesh, burr hole cover, etc.)	2 (3.17)
		osteoclastic without cranioplasty	1 (1.59)
		varies	7 (11.11)
7	Do you use fixed retraction during microvascular decompression?	yes, always or often	33 (52.38)
		no or only during the approach	25 (39.68)
		varies	5 (7.94)
8	Do you sacrifice the superior petrosal vein during microvascular decompression?	yes, generally or mostly	4 (6.35)
		yes, if necessary (visual obstruction, compression, etc.)	18 (28.57)
		no, never, or only in case of hemorrhage	41 (65.08)
9	How do you usually separate the nerve from the vessel during microvascular decompression?	Teflon	47 (74.60)
		muscle	6 (9.52)
		other material (PVA, Dacron, Gelaspon, etc.)	4 (6.35)
		transposition /sling retraction without interposition	6 (9.52)
10	Do you secure the interposition material with fibrin glue?	yes, always or mostly	40 (63.49)
		no, never or rarely	16 (25.40)
		sometimes	7 (11.11)
11	Do you pursue watertight dural suturing after microvascular decompression (regardless of the use of Tachosil, dural substitute, or similar)?	yes, absolutely	53 (84.13)
		no, not imperatively	10 (15.87)
12	Do you routinely place a lumbar drain for microvascular decompression?	yes, before surgery	3 (4.76)
		yes, after surgery	0 (0)
		no	60 (95.24)
13	Do you generally consider microvascular decompression for trigeminal neuralgia secondary to multiple sclerosis?	yes	9 (14.29)
		yes, if the MRI shows neurovascular contact	35 (55.56)
		no	19 (30.16)
14	Do you indicate microvascular decompression for trigeminal neuralgia if there is NO neurovascular contact on imaging?	yes	16 (25.40)
		sometimes, after shared decision-making with the patient	42 (66.67)
		no	5 (7.94)
